# Application of Recycled Ceramic Aggregates for the Production of Mineral-Asphalt Mixtures

**DOI:** 10.3390/ma11050658

**Published:** 2018-04-24

**Authors:** Wojciech Andrzejuk, Danuta Barnat-Hunek, Rafat Siddique, Bartosz Zegardło, Grzegorz Łagód

**Affiliations:** 1 Faculty of Economic and Technical Science, Pope John Paul II State School of Higher Education in Biała Podlaska, Sidorska Str. 95/97, 21-500 Biała Podlaska, Poland; w.andrzejuk@dydaktyka.pswbp.pl; 2 Faculty of Civil Engineering and Architecture, Lublin University of Technology, Nadbystrzycka Str. 40, 20-618 Lublin, Poland; d.barnat-hunek@pollub.pl; 3 Department of Civil Engineering, Thapar University, Patiala, Punjab 147004, India; siddique_66@yahoo.com; 4 Faculty of Natural Sciences, Siedlce University of Natural Sciences and Humanities, 14 B. Prusa Str., 08-100 Siedlce, Poland; bart.z@wp.pl; 5 Faculty of Environmental Engineering, Lublin University of Technology, 40B Nadbystrzycka Str., 20-618 Lublin, Poland

**Keywords:** mineral-asphalt mixtures, aggregate from sanitary ceramic wastes, environmentally friendly construction materials

## Abstract

This paper describes a method of designing and producing innovative mineral–asphalt mixtures, which utilize waste aggregate from the recycling of sanitary ceramics. The work presents the basic properties of the ceramic material, the investigation concerning the microstructure of the aggregate obtained from the grinding of waste, and a comparison with the images obtained for the aggregates usually employed in mineral–asphalt mixtures. The mixtures were designed for the application in the wearing course. Four series of mixtures were prepared. In the first and second, the ceramic aggregate constituted a partial substitute for dolomite, whereas in the third, we substituted granodiorite, and the fourth series contained only dolomite. The mixtures were examined for the content of soluble binder, the bulk density of samples, the presence of voids, the space filled with binder, and the susceptibility to water and frost corrosion. The obtained results were compared with the standard requirements. The microstructure as well as the contact zone in the considered mineral–asphalt mixtures are presented based on research conducted by means of a scanning electron microscope (SEM).

## 1. Introduction

The degradation of the natural environment observed in recent years, resulting from rapid development, has made ecological security one of the top issues for contemporary science to address. The increased production occurring in industry, which consumes a large quantity of energy and yields a huge amount of post-production waste, is especially unfavorable [1,2].

Moreno-Maroto et al. have assessed different wastes for lightweight aggregate (LWA) manufacturing: granite and marble sludge (COR), sepiolite rejections (SEP), and polyethylene–hexene thermoplastics (P) [3]. Some most recent studies on the application of wastes for LWA sintering were presented by Liu et al. [4]—involving sewage sludge and river sediments, Li et al. [5]—sewage sludge and saline clays, as well as Franus et al. [6]—concerning the changes in the microstructure that occurred when spent oil was added to LWA production. Smarzewski and Barnat-Hunek investigated the mechanical and durability-related properties of high-performance concrete made with coal cinder and waste foundry sand [7]. Apart from the re-use of a waste substance, the advantages include the reduced consumption of aggregates from natural deposits, as presented, e.g., in studies by Suchorab et al. [8]. The results of the research prove that this type of additive, regardless of the form in which it is used in a concrete mixture (powder or aggregate), deteriorates the strength of concrete. Miličević et al. studied the mechanical properties of concrete made with crushed clay bricks and roof tiles aggregate [9].

Ceramic materials are produced in abundance in various parts of the world; they are also exported to other regions owing to their durability and diversification, which makes them suitable for use in numerous designs [10]. Some studies present work conducted on concrete produced with the use of white ceramics [10,11,12]. Taking into account the properties of ceramics, this has the potential for application as a supplementary cement material or aggregate in conventional concrete [10,12,13]. Ceramic waste constitutes porous materials that act as a wet hardening agent for hydration of cement paste and reduce the autogenous shrinkage, finally increasing the mechanical strength [14]. Ceramic aggregate in concrete improves the layout of pores, reduces the volume of macropores, and increases the number of capillary pores. Zirconium found in ceramic materials does not migrate into cement paste or influence the chemical reaction of hydration [12]. These factors contribute to increasing the mechanical strength and durability to environmental, chemical, and physical factors [10,12].

In terms of ecologically hazardous substances, ceramic products are inert. According to the Regulation of the European Parliament and Council of 2006 on shipments of waste, sanitary ceramic waste is not classified as hazardous waste [15]. Their production process is irreversible. The ceramic products are persistent and non-biodegradable. The increasing demand for ceramic products results in a growing amount of landfilled waste. Due to the abovementioned reasons, many research institutes made an attempt to devise rational methods of recycling ceramic wastes. The main direction involves using the waste for concrete production. The conducted analyses indicate that ceramic is a durable material with good strength parameters, and its application for concrete mixtures does not require special treatment. The authors of studies confirm the usefulness of recycled aggregates for concrete production. This type of material, acquired from waste sanitary or technical ceramics (e.g., electrical insulators), has a beneficial effect on the properties of concrete [16]. The results show that the greater the ceramic aggregate addition is, the better the strength parameters of concrete are. The interesting properties of the concrete produced with broken sanitary ceramics are also presented by Halicka et al. [16]. In order to economically justify the performed works, the authors seek prospective special applications of this type of concrete. The research results indicate that it can be used in cases where concrete is exposed to high temperatures or in places where high abrasion resistance is required. A method of designing highly resistant concrete produced only with ceramic aggregate was presented in [17]. The analysis of results proved that, owing to the porous structure of aggregate granules, the contact point of cement stone with the aggregate is characterized by higher durability than traditional aggregates, and the concretes obtained in this way exhibit higher strength parameters.

Referring to the results of the abovementioned works, the authors of this paper made an attempt to produce mineral–asphalt mixtures that include waste ceramic aggregates. Few publications on this topic exist in the literature. The possibility of using the waste obtained from grinding ceramic roof tiles as a partial substitute for natural aggregates in the binding layers of mineral–asphalt mixtures was presented in [18]. On the basis of the conducted research, it was concluded that ceramic aggregates can be applied in asphalt concrete and their addition up to 30% was appropriate for roads with medium and low traffic intensity. The authors of the paper focused on designing the mixtures for improving the wearing course of roads. The mixtures were examined for: the content of soluble binder, bulk density of samples, content of voids, content of the spaces filled with binder and susceptibility to water and frost corrosion. The results were compared with the ones obtained for the reference samples prepared from traditional aggregates (the analysis of the available mineral–asphalt mixtures (MAM) research results based on the traditional aggregates). The results were also analyzed in terms of standard requirements.

## 2. Materials and Methods

### 2.1. Material Properties

The study materials were selected by sorting the ceramic waste deposited on a dump adjacent to an industrial plant producing sanitary ceramics, mainly washbasins with cracks, enamel damage, or uneven surface. The acquired waste was crushed in a jaw crusher. The crushers operated in a way that enabled us to sort the grains into two sizes, i.e., fine: 0–4 mm and coarse: 4–8 mm. Grains of larger size were crushed again.

It was planned that the obtained aggregate would partially substitute the aggregates traditionally employed in mineral-asphalt mixtures, i.e., dolomite and granodiorite. A series of experiments were conducted for the obtained and reference aggregates, corresponding to the investigations of the natural aggregates commonly used for concrete production, such as: specific density, bulk density, compressive strength, absorptivity and crushing degree [19,20,21,22]. The findings were compared with the literature data [23] for the most popular aggregates employed in road industry. The comparison of results was presented in Table 1.

**Figure 1:**
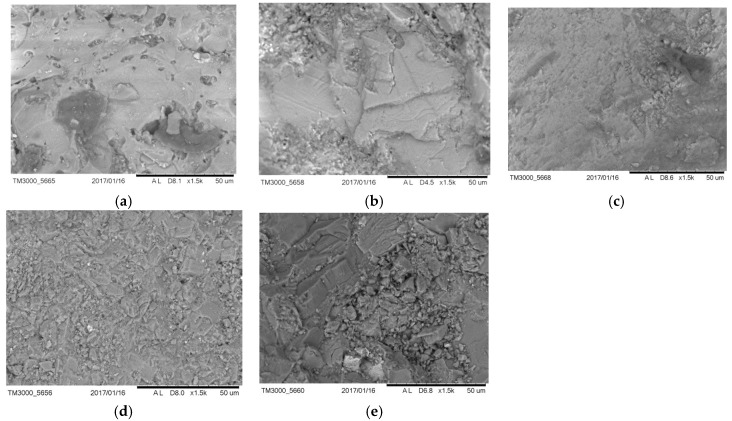
Images of aggregate grains structure: (**a**) obtained from sanitary ceramics, (**b**) dolomite, (**c**) granodiorite, (**d**) basalt, (**e**) sand (×1500).

Using a scanning electron microscope, an analysis of the surface structure of the ceramic and traditional aggregates was conducted, including dolomite, granodiorite, basalt and sand, which were the ingredients of mixtures, including the reference ones. The structure of the grain surface is presented in Figure 1. The microstructure of aggregate grains has a significant impact on the adhesion of the asphalt to the aggregate; the more developed the aggregate grain surface, the greater the contact area of asphalt, as described by Moraes et al. [24]. On the basis of the possible structural similarity of granodiorite (Figure 1c) and ceramics (Figure 1a), it can be supposed that the adhesion of asphalt to ceramic aggregate grains should not differ from the adhesion of asphalt to granodiorite. Detailed analyses are presented in point 5.

Road asphalt 50/70 with the parameters presented in Table 2 constituted the binder in the mineral-asphalt mixture.

Limestone dust was applied as a filling aggregate for bituminous mixtures and surface dressing used on roads, airports and other surfaces intended for traffic, with the grain size of 2.69 g/cm^3^ and properties presented in Table 3.

### 2.2. Designing Method of the Mixture

Before the design of the mineral–asphalt mixture could begin, the requirements laid down in WT-2 2014—part I, Mineral–Asphalt Mixtures, Technical Requirements were assumed [25]. The program involved creation of a mixture intended for the wearing course (WC). Preparation of four mixture series was proposed. The series 1 and 2 mixtures (WC-1, WC-2) were prepared on the basis of dolomite and ceramic aggregate, whereas the series 3 mixture (WC-3) was based on granodiorite and ceramic aggregate. The series 4 mixture (WC-4) constituted a reference mixture and is solely comprised of the dolomite aggregate. It was assumed that the laboratory recipe for the WC would be prepared for the asphalt concrete intended for a wearing course, with a grain size of 0–11.2 mm, for the surfaces with traffic load 1 ÷ 2 (TL1 ÷ 2). The preparation of the recipe was based on the standards [26,27]. The first stage of the design involved the choice of appropriate graining. It was planned that the total grain composition of the aggregate would include limestone dust, added as a filler. Table 4 presents the established aggregate compositions for consecutive series. Sieve analyses were conducted for these grain compositions and initial graining curves were prepared.

The aggregates deposited on the premises of bituminous mass production plant are dusty. The presence of dust negatively impacts the production properties of mineral-asphalt mixtures; hence, the production plants employ the dedusting process.

**Figure 2:**
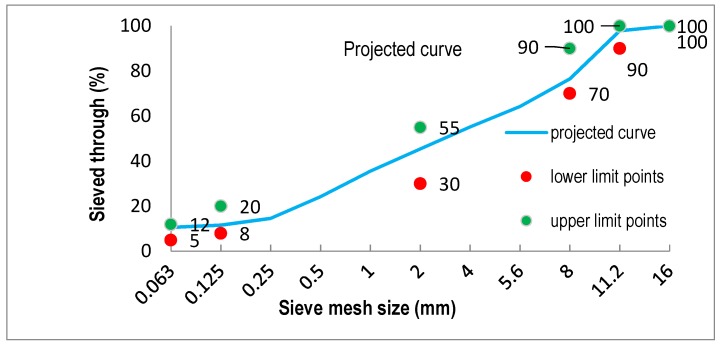
The graining curves for WC-1.

This process enables us to partially remove dust from aggregates. All aggregates, except for limestone dust, undergo dedusting. This is because limestone dust is supplied directly into the mixer, skipping the dedusting process. The amount of dust removed depends on the type of a production plant as well as the humidity. Dedusting causes a change in the aggregate grain size, which needs to be taken into account during the design stage of a mixture. The dust removal level assumed in the recipe amounted to 50%, being most common in MAM production plants. Hence, it was necessary to recalculate the sieving. While determining the content of particular aggregates in mineral mixture (MM), one should remember to select proportions so that the graining curve is between the borderline curves, in accordance with [25]. The comparison result was positive. The graining of all analyzed series in both batches was between the borderline curves. Because the series were nearly the same, a single exemplary graph is presented (Figure 2).

In order to determine the amount of asphalt in a mixture, a calculation method accounting for the specific surface area of mineral mixture of aggregates and the thickness of the asphalt film was selected [28]. As far as the production of mineral-asphalt mixtures is concerned, it is vital that the grains should be covered with a film of asphalt binder of appropriate thickness. If the film is too thin, the mixture will segregate and lose its properties. The thickness of the asphalt film is dependent on the type of binder and the specific surface area of a mineral mixture, which was calculated by means of Equation (1):

(1) $$F=(0.04g+0.06z+0.10s+1.5f)\frac{2.65}{\rho_{a}}$$

where:

*F*—specific surface area of the designer mineral mixture, (m^2^/kg)
*g*—content of the fraction >4 mm, (%)
*z*—content of the fraction >0.25 ≤ 4 mm, (%)
*s*—content of the fraction >0.063 to ≤0.25 mm, (%)
*f*—content of the fraction ≤0.063, (%)
*ρ_a_*—density of the mineral mixture, (g/cm^3^)

Knowing the specific surface area, the required content of asphalt for a mineral mixture was determined [28]:

(2) $$A_{k}=\frac{F\;b\;\rho_{asf}}{10}\;[\%]$$

where:

*A_k_*—content of binder in relation to the mineral mixture, (%)
*F*—specific surface area of the mineral mixture, (m^2^/kg)
*B*—thickness of bituminous film, (μm)
*ρ_asf_*—binder density, (g/cm^3^)

The thickness of asphalt film, in line with [29] should assume the type of utilized asphalt and the specific surface area of the mineral mixture.

Depending on the type of applied asphalt, the thickness of film for various kinds of asphalt should amount to:

- 35/50 asphalt—3.2–2.7 μm
- 50/70 asphalt—2.8–2.4 μm
- 70/100 asphalt—2.6–2.2 μm
- 100/150 asphalt—2.2–1.7 μm.

The recommended thickness of the asphalt film depending on the specific surface area of the mixture is presented in Table 5.

The knowledge on the composition of mineral mixtures and the amount of asphalt in MAM enabled to determine the final composition of mineral-asphalt mixtures for all series. The results were presented in Table 6.

The preparation of the samples for studies was divided into several stages. Stage 1 constituted the preparation of the aggregate, which involved weighing particular aggregate batches with at least 10% additions, drying them to constant mass, leaving them to cool down, sieving out dust in line with the assumption of the laboratory recipe, weighing the particular aggregate graining in accordance with that calculated for the batch, and thermostating in a dryer at 155 ± 5 °C for 8 h. Stage 2 consisted of the preparation of asphalt in the following way: the asphalt was poured into a container and placed in a dryer so that thermostating of the aggregate and asphalt would finish at the same time.

Afterwards, the ingredients were mixed manually. The mixing process was conducted in the following way: a vessel was placed and heated on a heating plate; weighed aggregate and binder were subsequently poured and mixed with stirring motion. The next stage involved injecting an appropriate amount of asphalt and mixing the batch until the aggregate was entirely covered with asphalt film, for no longer than 5 min.

### 2.3. Methods

The first of the planned investigations involved determining the binder content and graining of particular aggregates, including filler, an in the mixture. The investigation included an extraction performed on a sample of a mineral–asphalt mixture, i.e., subjecting the sample to a solvent—tetrachloroethylene. These operations resulted in the separation of the binder from the aggregate and the separation of the filler. The study aims to identify the differences in the amount of asphalts injected to the batch and the amount calculated after extraction. The differences usually stem from the presence of insoluble asphalt absorbed by aggregate grains and insoluble particles in the applied solvent.

The experiment was conducted on the samples collected with the standard [30], where the minimal mass of a sample for a mineral–asphalt mixture with a grain size up to 11.2 mm was determined as 300 g. In order to conduct the experiment, the following devices were prepared: an extractor, a vortex mixer with a set of sieves, a fan dryer enabling to maintain the temperature of 110 ± 5 °C, and laboratory scales.

The investigation for each series was initiated by weighing empty and filled (with the analytic sample) extractor tubes. Afterwards, a paper filter was applied on a dried extraction thimble and everything was weighed again. Subsequently, the extraction thimble and tube were placed in an extractor and the device was turned on. When the extraction was finished, the extraction thimble with the filler was cooled and weighed. The tube with aggregate was put into a dryer at 110 °C and stored until it was totally dry. Then, aggregate was extracted and cooled, weighed, and sieved through the prepared set of sieves with the following mesh sizes: 0; 063; 0.125; 0.25; 0.5; 1; 2; 4; 5.6; 8; 11.2 mm. The content of asphalt in percent was calculated from the mass difference by means of Equation (3) [31].

(3) $$S=\frac{M_{m}-[M_{k}+(m_{2}-m_{1})]}{M_{m}}\times 100\%$$

where:

*M_m_*—mass of the mineral-asphalt mixture, (g)
*M_k_*—mass of aggregate excluding the filler in the extraction thimble, (g)
*m*_1_—mass of the extraction thimble with filter prior to extraction, (g)
*m*_2_—mass of the extraction thimble with filter after extraction, (g).

So-called Marshall samples were prepared for further experiments. The following devices were used for this purpose: a thermostat-equipped fan dryer, a heating plate for manual mixing, laboratory scales, a thermometer capable of measuring temperatures up to 300 °C with the accuracy of at least ± 2 °C, a compacting mold with an internal diameter of 101.6 ± 0.1 mm, a Marshall compactor, and small support equipment: bowls, pots, spatulas, cleaning cloth, etc.

The samples were prepared in accordance with [32]. The weighed amount of MAM should be adjusted so that the sample (after compacting) has the following dimensions: diameter (101.6 ± 0.1) mm and height (63.5 ± 2.5) mm. Usually, the weighed amount for a single sample ranges between 1150 g and 1250 g. The amount of mineral mixture weighed beforehand should be placed in a suitable vessel in a dryer and heated to the temperature established in the assumptions for the preparation of a mineral–asphalt mixture. The aggregate can be placed in a single or several vessels. In the case of 50/70 asphalt, the mixture temperature ranges from 140 to 180 °C. The aggregate placed into a dryer should be left for at least 8 h. The weighed amount of binder should be dried for an hour in a separate vessel. The asphalt should be placed in a dryer in such a way that the thermostating of the aggregate and asphalt would finish at the same time. The aggregate can undergo thermostating for a longer time, but this is not the case with asphalt. Wetfix adhesive is added to the asphalt, in line with the recipe. The ingredients are mixed manually. A vessel with handles, e.g., a pot with appropriate volume in relation to the batch size, is placed on the heating plate and heated. The aggregate is poured into the pot and stirred. Afterwards, asphalt is added very carefully, because adding too much would require repeating the entire process. The content is mixed until all MM grains are covered with asphalt and the MAM becomes homogenous. The maximum mixing time, which in this case equals 5 min, should not be exceeded. Prior to forming samples, the molds, along with the equipment, should be placed in a dryer at 120–130 °C. A paper separator is placed in the mold, removed from the dryer, and subsequently filled with the weighed amount of MAM; it is then evened out and another paper separator is added. Prior to compacting, the piston needs to be heated. The prepared mold is placed in a Marshall compactor and compacted. The number of blows depends on the intended MAM application. In this case, it equals 50 blows. After the compaction, a sample is turned 180° and subjected to another 50 blows. The experiment can be initiated 4 h after a sample was removed from the mold, when it cools enough not to be damaged.

The aim of the first experiment, conducted on the samples prepared in such way, was to determine the density of a mineral–asphalt mixture, which is the quotient of the sample mass and its volume, without voids. The experiment was performed on the samples prepared in accordance with the standard [33], which stated that the mass of an analytic sample in grams should be at least 50 times greater than the largest coarse grains of the aggregate in the mineral–asphalt mixture. The samples were crushed into individual grains or lumps so that the diameter did not exceed 6 mm. In the case of the compacted sample, prior to compacting, it was placed in the dryer at 110 °C and heated until compaction was possible. In order to conduct the experiments, the following equipment was prepared: laboratory scales, a fan dryer with a thermostat, a vacuum generator, a pycnometer, a water bath, and a spatula.

The pycnometer was calibrated prior to the experiment. Afterwards, an empty pycnometer was weighed for each series. Then, a crushed mixture sample was poured into the pycnometer to the 2/3 of its capacity, covered with the head and weighed. The head was subsequently removed, distilled water was added to the level of 3 cm below the pycnometer bottle neck, two drops of an active surfactant were added and the pycnometer was placed in a vacuum chamber. The trapped air was removed under vacuum pressure (4 kPa) for 15 min. Afterwards, with the head placed again, the water was topped up to slightly below the line on the bottle neck. The pycnometer was then placed in a water bath at 25 °C for 2 h. When the pycnometer was removed from the bath, the water was topped up again to the line on the neck, surface dried, and weighed. The density *ρ_mw_* of the mineral–asphalt mixture, expressed in g/cm^3^ was calculated according to Equation (4) [23]:

(4) $$\rho_{mw}=\frac{m_{2}-m_{1}}{V_{p}-(m_{3}-m_{2})/\rho_{w}}$$

where:

*V_p_* —pycnometer volume, (cm^3^)
*ρ_w_*—density of distilled water assumed for the temperature of the experiment, (g/cm^3^)
*m*_1_—mass of the pycnometer with the head, (g)
*m*_2_—mass of the pycnometer with the head and mineral-asphalt sample, (g)
*m*_3_—mass of the pycnometer with the head, sample and distilled water, (g).

The next experiment involved determining the bulk density of the mineral–asphalt mixture, which constitutes the quotient of the sample mass and its volume with voids.

The experiment was conducted on the Marshall samples, prepared as described above. Conducting the experiment required preparation of the following instruments: laboratory scales, thermometer, water bath, and calipers. Prior to the investigation, the samples were cleaned by brushing loose grains off the sample. Afterwards, each sample was weighed and placed in the water bath for 40 min. When the samples were soaked, they were placed in a basket submerged in water and suspended under a hydrostatic balance so the mass of the water-soaked sample could be read. Then, the temperature of water in the water bath was measured in order to determine the density of water. The sample was then removed from water, surface dried, and weighed. The bulk density in the saturated, surface dry state of the mineral–asphalt mixture expressed in g/cm^3^ was calculated with Equation (5):

(5) $$\rho_{bssd}=\frac{m_{1}}{m_{3}-m_{2}}\times \rho_{w}$$

where:

*ρ_w_*—density of distilled water assumed for the temperature of the experiment, (g/cm^3^)
*m_1_*—mass of a dry sample, (g)
*m_2_*—mass of a sample saturated with water, (g)
*m_3_*—mass of a surface dry, saturated sample, (g).

Afterwards, the presence of voids in the mineral–asphalt mixture, which are the free, air-filled spaces between the aggregate grains covered with a binder film in the compressed samples, was determined. This is calculated on the basis of the bulk density of a mineral–asphalt mixture as well as the density of the mineral–asphalt mixture.

Indication of voids was carried out in line with the methodology presented in the standard [34]. Voids *V_m_*, expressed in %, were calculated by means of Equation (6) [24]:

(6) $$V_{m}=\frac{\rho_{m}-\rho_{b}}{\rho_{m}}\times 100\%$$

where:

*V_m_*—content of voids in a sample of the mineral-asphalt mixture up to 0.1%,
*ρ_m_*—density of the mineral-asphalt mixture, (g/cm^3^)
*ρ_b_*—bulk density (g/cm^3^).

Afterward, the content of voids in the mineral mixture filled with binder was calculated. This compares the asphalt volume in the mineral–asphalt mixture to the content of voids in the compacted mineral mixture, which is calculated on the basis of the binder and void content in MM as well as the bulk density of a given sample along with the binder density.

The content of voids in MM filled with asphalt is provided with an accuracy of 0.1% and calculated on the basis of Equation (7), in line with the standard [34]:

(7) $$VFB=\frac{B\times \frac{\rho_{b}}{\rho_{B}}}{VMA}\times 100\%$$

where:

*VMA*—corresponds to the content of voids in the mineral mixture, expressed in %, which can be calculated by means of the Equation (8) [24],

(8) $$VMA=V_{m}+B\cdot \frac{\rho_{b}}{\rho_{B}}$$

where:

*B*—content of binder in the mineral-asphalt mixture, (%)
*ρ_b_*—bulk density of a mineral-asphalt mixture sample, (g/cm^3^)
*ρ_B_*—binder density, (g/cm^3^)
*V_m_*—content of voids in the mineral-asphalt mixture, (%).

The aim of the final experiment was to determine the resistance of the mineral-asphalt mixture samples to the effect of water and frost, expressed by means of ITSR (indirect tensile strength ratio) index. This index involves the ratio of the indirect tensile strength obtained on conditioned (wet) samples to the indirect tensile strength of the dry samples.

The experiment was conducted on Marshall samples, in accordance with the standard [35]. The following instruments were prepared: Marshall compactor characterized with the parameters stated in the standard [36], test head conforming to the standard [35], vacuum pump, vacuum tank, water bath, thermostatic chamber, freezer, scales, thermostat-equipped dryer, calipers, plastic bags, and a syringe.

In order to determine the susceptibility to the water and frost effect, 10 samples were prepared for each series. The samples for research were compacted according to the guidelines found in [26]. Each side of a sample was hit with 35 blows. The dimensions of each sample were measured and the bulk density was calculated. A set of 10 samples was divided into two equal parts with similar dimensions and bulk density. The first group of similar samples constitute the “wet” samples, whereas the other—the “dry” ones. The samples from the “dry” set were subjected to conditioning by storage on a level surface at room temperature (20 ± 5°C). The set of “wet” samples was placed in a vacuum apparatus filled with distilled water. The set pressure was maintained for (30 ± 5) min. Afterwards, the samples were placed in the water bath at (40 ± 1 °C) for 68 to 72 h. The samples were subsequently removed from the water bath and their surface was gently dried. The next step involved placing the samples in plastic containers, so that the surface fitted closely to the bag. The samples were then placed in a freezer at −18 ± 3 °C for approximately 16–18 h. After their removal from the freezer, the samples were once again put in the water bath at the temperature of 60 ± 1 °C. Following the thawing process, the samples were removed from the bags as quickly as possible. The samples taken from the water bath were stored at room temperature for at least two hours. The research procedure itself begins with achieving a temperature of 25 ± 2 °C for both the wet and dry sample sets. The experiment on a sample should be carried out 1 min following the removal from water.

The ITSR expressed in % was calculated by means of the Equation (9), in line with the standard [35].

(9) $$ITSR=\frac{ITS_{w}}{ITS_{d}}\times 100\%$$

where:

*ITS_w_*—stands for the average strength of the wet samples, rounded up to an integer, which can be calculated from Equation (10) [35],

(10) $$ITS_{w}=\frac{2\times P_{w}}{\pi \times D\times H}$$

*ITS_d_*—corresponds to the average strength of the dry samples, rounded up to an integer, which can be calculated from Equation (11) [35],

(11) $$ITS_{d}=\frac{2\times P_{d}}{\pi \times D\times H}$$

where:

*P_w_*—maximum value of the compressive strength for the wet samples (kN)
*P_d_*—maximum value of the compressive strength for the dry samples (kN)
*D*—sample diameter rounded up to 0.1 (mm),
*H*—sample height rounded up to 0.1 (mm).

### 2.4. Scanning Electron Microscopy with EDS Analysis

SEM (Quanta FEG 250 microscope by FEI, Hillsboro, OR, USA), equipped with a system for the chemical composition analysis based on the energy-dispersive X-ray spectroscopy (EDS) manufactured by EDAX (Mahwah, NJ, USA), was used for determining the morphology and porous structure of MAM, and the interfacial transition zone between aggregates and asphalt.

## 3. Results

The results obtained from the research on the asphalt content following extraction are presented in Table 7.

Following the extraction, it was observed that there are differences in the amount of asphalt added to the batch and the amount of asphalt calculated after extraction. These differences resulted from the presence of undissolved asphalt absorbed by aggregate grains and particles undissolved in the solvent. In the first and second series, this difference amounted to 0.2% and was twice as high as the one in the third series, which equaled 0.1% According to the standard [37], these differences should not exceed ± 0.5%. Therefore, the designed mixtures conform to the abovementioned requirements. The graining of the aggregate produced after extraction is presented in Table 8, Table 9 and Table 10.

The experiment showed that the graining of the aggregate differs between the ones introduced to the batch and the ones obtained after extraction. According to the standard [37], the differences should be within range of acceptable deviations, which amount to:

−8 ± +5 for the aggregates stopped on the 11.2 mm sieve,
±7 for the aggregates stopped on the 5.6 mm sieve,
±6 for the aggregates stopped on the 2.0 mm sieve,
±4 for the aggregates stopped on the 0.125 mm sieve,
±2 for the aggregates stopped on the 0.063 mm sieve.

**Figure 3:**
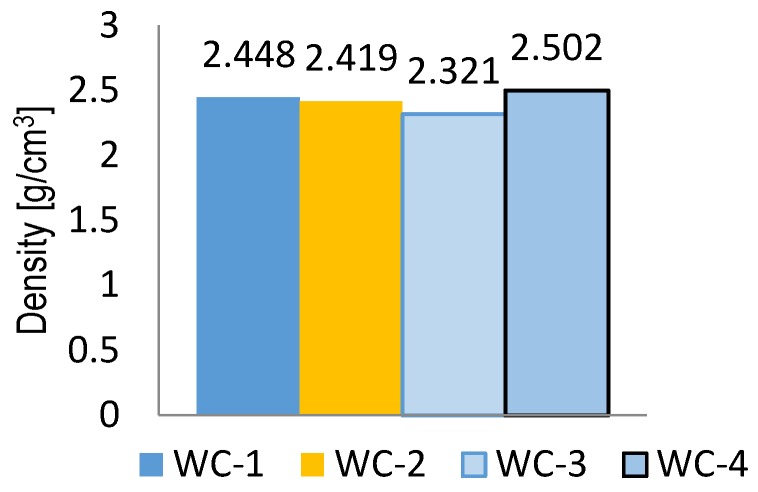
Density of mineral-asphalt mixtures.

**Figure 4:**
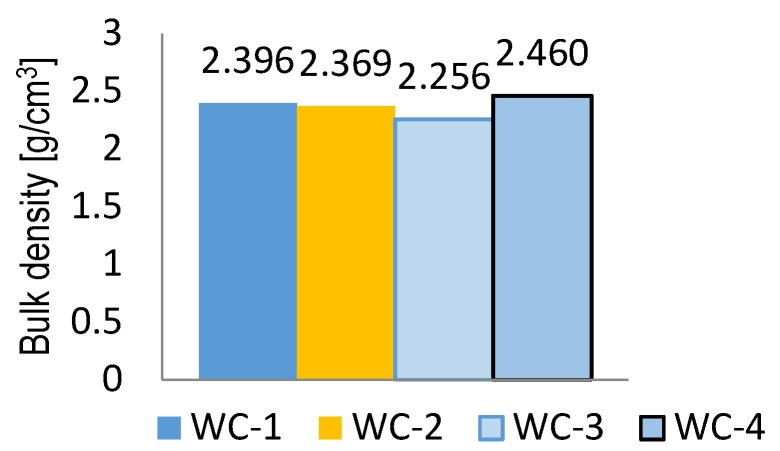
Bulk density of mineral-asphalt mixtures.

The density values obtained for particular series were presented in Figure 3. The WC-3 sample was characterized by lower density—equal to 2.321 g/cm^3^—than the samples prepared with dolomite. A similar situation concerned the density of the aggregates used for the preparation of mixtures. The granodiorite aggregate was characterized by a lower density than the dolomite aggregate. The bulk density values for particular series are presented in Figure 4.

A sample of mineral-asphalt mixture prepared with granodiorite was characterized by a lower bulk density, amounting to 2.256 g/cm^3^, than the samples prepared with dolomite.

The void content calculations results in a mineral-asphalt mixture for particular series are presented in Table 11.

The mineral-asphalt mixtures prepared with granodiorite (WC-3) reached the highest content of voids, amounting to 2.8%. In the case of the mixtures prepared solely with dolomite (WC-4), voids reached the lowest values and were 39% lower than WC-3 MAM. Therefore, it may be supposed that due to the higher absorptivity of the ceramic aggregate presented in Table 1, a partial absorption of asphalt by the waste aggregate occurred. A similar situation was described by Silvestre et al. [18] and Pérez et al. [38]. This caused an increase of voids in MAM.

According to [27], the voids in a mineral-asphalt mixture should not be lower than 1% and greater than 3%. The designed mixtures meet the presented technical requirements.

The results of void content calculations in a mineral mixture filled with binder for particular series are depicted in Table 12.

According to [27], the voids in the mineral mixture should reach at least 14%. On the other hand, the voids in the mineral mixture filled with binder should not be lower than 75% and greater than 93%. Therefore, the designed mixtures fulfil the technical requirements.

The results of indirect tensile strength ratio (ITSR) calculation were presented in Table 13.

**Figure 5:**
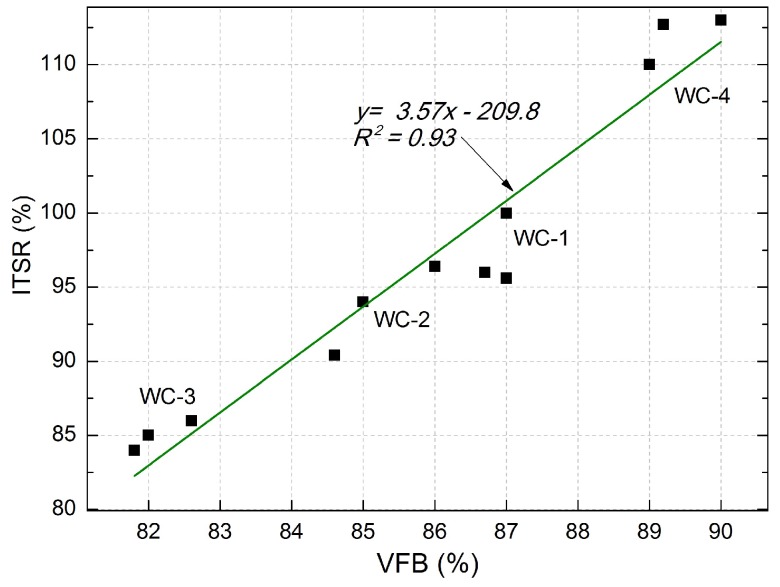
Correlation between voids in the mineral mixture filled with VFB binder and indirect tensile strength ratio (ITSR).

In accordance with [27], the indirect tensile strength ratio for the mineral-asphalt mixture for the wearing course should be at least 90%. The WC-3 mixture does not meet this criterion, but the remaining three mixtures do. The highest ITSR was obtained with WC-4 MAM and it was 24% higher than the value achieved with WC-3. The best results were obtained with the mixtures containing dolomite alone and the mixture comprising dolomite and ceramic aggregate with the graining of 0/4. A significant influence of voids in MM filled with binder (VFB) on the ITSR was observed (Figure 5).

**Figure 6:**
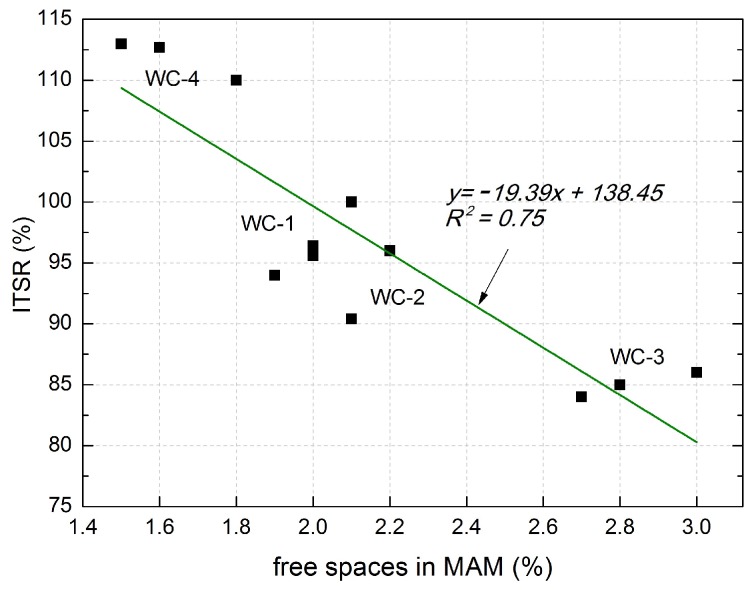
Correlation between the voids in MAM and ITSR.

The correlations can be described using the equation: *y* = 3.57*x* − 209.8, which is characterized by a good coefficient of determination *R*^2^ = 0.93. The higher the void content in MM, the higher the ITSR value. The type of utilized aggregate also influences ITSR, with dolomite aggregate yielding the best results. The resistance to the effect of water and frost characterizing MAM also depends on the content of voids (Table 11). An inverse dependency was observed in this case (Figure 6). The higher the void content in MAM, the lower the tensile strength—which is the parameter corresponding to frost resistance. The linear trend exhibited the coefficient of correlation *R*^2^ = 0.75 and relatively low errors in the intercept.

It was observed that a mixture comprising granodiorite and waste ceramics is least resistant to the water and frost effect among the analyzed mixtures intended for use in the wearing course. 

### Scanning Electron Microscopy with EDS Analysis

**Figure 7:**
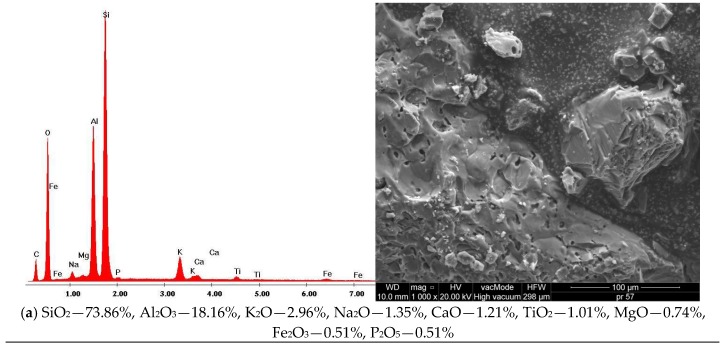

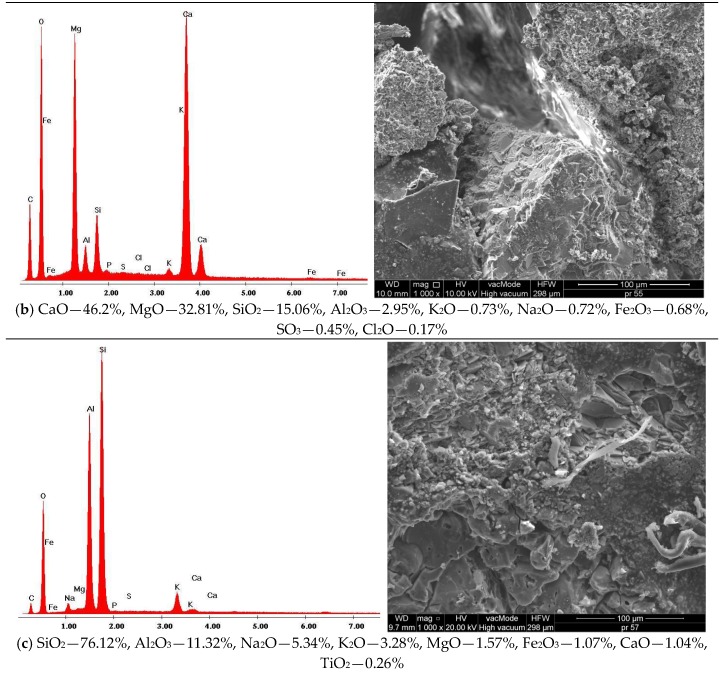
SEM Microstructure of MAM, results of the elemental analysis in EDS microarea: (**a**) WC-1, (**b**) WC-4, (**c**) WC-3.

The MAM microstructure examination, coupled with the EDS analysis, was conducted to determine the morphology and texture of the created forms as well as identify the basic chemical components of MAM (Figure 7).

**Figure 8:**
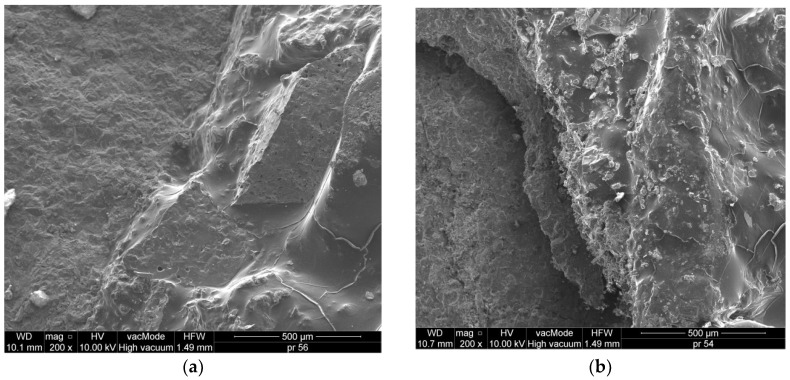

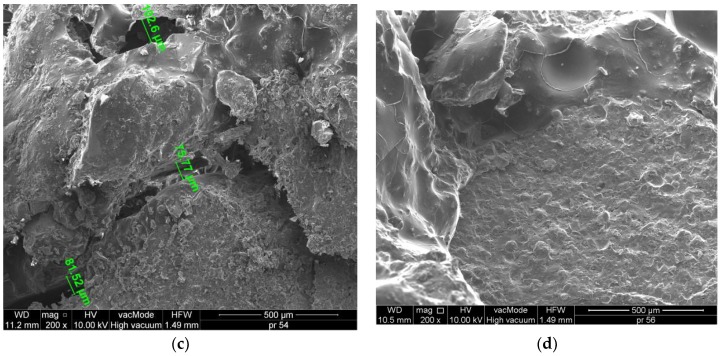
Micro-structure of MAM: (**a**) WC-1 dolomite + ceramics 0/4, (**b**) WC-2 dolomite + ceramics, (**c**) WC-3 granodiorite + ceramics, (**d**) WC-4 dolomite (×200).

The lack of cracks or air gaps, further supplemented by good adhesive properties between the ceramic aggregate (Figure 8a,b), dolomite aggregate (Figure 7d) and asphalt, resulted in the high durability and frost resistance of WC-1, WC-2 and especially WC-4. The cracks and poor adhesion between the granodiorite (Figure 7c) and asphalt could have influenced the increase of voids in MAM, as well as a drop in durability and frost resistance of this MAM, which was confirmed by earlier studies. There are 75–102.6 µm cracks in the mixture, which results in lower tensile strength and the lowest density among the analyzed MAMs, equal to 2.256 g/cm^3^.

In Figure 8, the chemical composition of the MAM is presented on the basis of energy dispersive spectrometry (EDS). A similar chemical composition of WC-1 and WC-3, with the dominant content of SiO_2_ and Al_2_O_3_, was observed. The chemical composition of the mixture with dolomite (WC-4) is different, because CaO and MgO are the prevailing compounds. The content of silica in this mixture is approximately 5 times lower (15.06%).

## 4. Conclusions

The presented research results prove that the ceramic aggregate is not suitable for all types of mineral-asphalt mixtures. The performed studies, conducted on the basis of the required technical specification [27] indicated that:

a. The obtained results of soluble binder content and aggregate produced after the extraction, conducted for the mixtures in each series, are comparable for typical mineral-asphalt mixtures and do not exceed the standard values.
b. The study on the density and bulk density of the mineral-asphalt mixture, on the basis of which the content of voids in the mineral-asphalt mixture and the mineral mixture filled with binder were calculated, indicates that these parameters are within the ranges of the standard requirements. The ceramic aggregate addition increased the void content in MAM by 15% through increased asphalt absorption and porous structure of the material.
c. The microstructure investigation indicated a very good adhesion of asphalt to the carbonate and ceramic aggregate. On the other hand, wide cracks appeared in the mixture with granodiorite, increasing the void content in MAM and reducing the bulk density of the mixture.
d. The highest resistance to water and frost was obtained in the mixture with dolomite WC-4 as well as dolomite and waste sanitary ceramics aggregate WC-1 and WC-2. The highest ITSR was obtained for WC-4 MAM and it was 24% higher than ITSR for WC-3 mixture.
e. A very good correlation between the content of voids in the MM filled with binder, as well as void content in MAM and the ITSR – corresponding to the resistance to water and frost – was obtained.
f. The conducted research indicates that ceramics may be successfully applied in the asphalt mixtures intended for use in the wearing course based on carbonate aggregates, i.e., dolomite. The mixture with 20 and 30% addition of recycled ceramic aggregate, used as a partial substitution of the natural dolomite aggregate, meets the majority of requirements related to mechanical properties stated in the technical specification.

## Figures and Tables

**Table 1:** Properties of ceramic aggregate in relation to aggregates used in concretes [16].

Property	Granite	Granodiorite	Porphyry	Diabase	Basalt	Quartzitic Sandstone	Compact Limestone	Dolomite	Ceramic Aggregate
Specific density (kg/dm^3^)	2.3–2.8	2.67	2.5–2.6	2.8–2.9	2.6–3.2	2.6–2.7	2.6–2.9	2.4–2.8	2.64
Bulk density (kg/dm^3^)	2.1–2.7	2.62–2.64	2.3–2.4	2.6–2.8	2.5–3.1	2.4–2.6	2.5–2.8	2.2–2.6	2.36
Compressive strength (MPa)	160–240	185–215	160–300	180–250	250–400	120–200	80–180	60–180	400–600
Modulus of elasticity (GPa)	13–61	-	36–68	70–90	56–99	4–43	21–53	18–48	40–70
Thermal expansion coefficient (α∙10^−6^)	5.0–9.0	6.0–9.0	7.0–9.0	7.0–9.0	8.0–12.0	12.0–18.0	1.0–8.0	3.0–12.0	6.0–7.0
Absorptivity (%)	0.2–0.5	0.34–0.47	0.2–0.7	0.1–0.3	0.1–0.4	0.2–0.5	0.3–1.5	0.3–2	1.53
Crushing degree (%)	18	14.9	13	16	3.8	15	18-20	20	8.9

**Table 2:** Parameters of road asphalt 50/70.

Parameter	Unit	Value
Penetration at 25 °C	1/10 mm	50–70
Softening point	°C	46–54
Embrittlement temperature	°C	≤−8
Ignition temperature	°C	≥230
Solubility	% m/m	≥99.0
Mass change (absolute value)	% m/m	≤0.5
Remaining penetration at 25 °C	%	≥50
Softening point increase	°C	≤9

**Table 3:** Parameters of limestone dust.

Property	Unit	Mean Value	PN-EN 13043:2004 Standard Requirements
passing % through:			
0.150 mm	%	94.5	-
0.125 mm	%	91.7	85–100
0.075 mm	%	81.9	-
0.063 mm	%	76.8	70–100
CaCO_3_ content	%	91.6	≥90
Humidity	%	0.2	≤1.0

**Table 4:** Composition of mineral mixtures for series 1–4.

No.	Mixture Ingredients	WC-1	WC-2	WC-3	WC-4
1	Filler	limestone	limestone	limestone	limestone
2	Fine-grained aggregate	dolomite 0/2	quartz 0/2 dolomite 0/2	-	quartz 0/2 dolomite 0/2
3	Coarse-grained aggregate	dolomite 2/8 dolomite 8/11	dolomite 2/8 dolomite 8/11 ceramics 4/8	ceramics 4/8 granodiorite 8/11	dolomite 2/8 dolomite 8/11
4	Aggregate with continuous granulation	ceramics 0/4	ceramics 0/4	ceramics 0/4 granodiorite 0/4	-

**Table 5:** Thickness of the asphalt film depending on the specific surface area.

The Specific Surface Area of the Mixture (m^2^/kg)	Thickness of the Asphalt Film b (μm)
0.023–1	40–80
1–3	15–40
3–5	6–15
5–10	4–10
10–25	2–8
25–50	1.5–4

**Table 6:** Composition of mineral and mineral-asphalt mixtures.

Material	WC-1		WC-2		WC-3		WC-4	
	% Share in MM	% Share in MAM	% SHARE in MM	% Share in MAM	% Share in MM	% Share in MAM	% Share in MM	% Share in MAM
limestone	9.0	8.5	9.0	8.3	9.0	8.5	6.0	5.6
quartz 0/2	-	-	16.0	15.0	-	-	22.0	20.7
dolomite 0/2	22.0	20.7	10.0	9.5	-	-	22.0	20.7
dolomite 2/8	24.7	23.3	10.0	9.5	-	-	30.0	28.3
dolomite 8/11	23.7	22.4	25.0	23.6	-	-	20.0	18.9
granodiorite 0/4	-	-	-	-	24.0	22.6	-	-
granodiorite 8/11	-	-	-	-	20.0	18.8	-	-
ceramics 0/4	20.6	19.3	15.0	14.1	25.0	23.6	-	-
ceramics 4/8	-	-	15.0	14.2	22.0	20.7	-	-
asphalt 50/70	-	5.8	-	5.8	-	5.8	-	5.8
TOTAL	100.0	100.0	100.0	100.0	100.0	100.0	100.0	100.0

**Table 7:** Results pertaining to the soluble binder content.

	WC-1	WC-2	WC-3	WC-4
Binder content determined on the basis of research (%)	5.6	5.6	5.7	5.6
Binder content according to the recipe (%)	5.8	5.8	5.8	5.8
Difference between the measured value and the recipe (%)	0.2	0.2	0.1	0.2
Acceptable difference (%)	±0.5	±0.5	±0.5	±0.5

**Table 8:** Aggregate graining following extraction for WC-1.

Sieve #, mm	WC-1				Acceptable Difference in the Composition of a Single Sample According to [37], %	Acceptable Difference in the Composition of a Single Sample According to WT-2 2008 [26], %
	Stopped on Sieve, %	Passed Through Sieve, %		Difference in the Composition, %		
		According to Extraction	According to the Assumed Composition			
16.0	0.0	100.0	100.0	0.0		
11.2	1.3	98.7	98.7	0.0	−8 ± +5	-
8.0	20.4	78.3	79.5	−1.2		
5.6	10.0	68.3	69.5	−1.2	±7	-
4.0	6.7	61.6	62.3	−0.7		
2.0	16.0	45.6	47.5	−1.9	±6	-
0.125	33.7	11.9	12.4	−0.5	±4	-
0.063	2.2	9.7	9.8	−0.1	±2	-
	<0.063 mm	9.7	9.8	−0.1	-	±3
	<0.125 mm	11.9	12.4	−0.5	-	±4
	0.063–2.0 mm	35.9	37.7	−1.8	-	±8
	≥2.0 mm	54.4	52.5	1.9	-	±8
	≥11.2 mm	1.3	1.3	0.0	-	−8 ± +5

**Table 9:** Aggregate graining following extraction for WC-2.

Sieve #, mm	WC-2				Acceptable Difference in the Composition of a Single Sample According to [37], %	Acceptable Difference in the Composition of a Single Sample According to WT-2 2008 [26], %
	Stopped on Sieve, %	Passed Through Sieve, %		Difference in the Composition, %		
		According to Extraction	According to the Assumed Composition			
16.0	0.0	100.0	100.0	0.0		
11.2	3.3	96.7	97.9	−1.2	−8 ± +5	-
8.0	22.8	73.9	76.5	−2.6		
5.6	11.4	62.5	64.3	−1.8	± 7	-
4.0	8.4	54.1	55.2	−1.1		
2.0	9.5	44.6	45.4	−0.8	± 6	-
0.125	33.7	10.9	11.6	−0.7	± 4	-
0.063	1.2	9.7	10.6	−0.9	± 2	-
	<0.063 mm	9.7	10.6	−0.9	-	±3
	<0.125 mm	10.9	11.6	−0.7	-	±4
	0.063–2.0 mm	34.9	34.8	0.1	-	±8
	≥2.0 mm	55.4	54.6	0.8	-	±8
	≥11.2 mm	3.3	2.1	1.2	-	−8 ± +5

**Table 10:** Aggregate graining following extraction for WC-3.

Sieve #, mm	WC-3				Acceptable Difference in the Composition of a Single Sample according to [37], %	Acceptable Difference in the Composition of a Single Sample According to WT-2 2008 [26], %
	Stopped on Sieve, %	Passed Through Sieve, %		Difference in the Composition, %		
		According to Extraction	According to the Assumed Composition			
16.0	0.0	100.0	100.0	0.0		
11.2	0.4	99.6	99.7	−0.1	−8 ± +5	-
8.0	11.8	87.8	89.2	−1.4		
5.6	20.0	67.8	71.3	−3.5	±7	-
4.0	13.0	54.8	57.0	−2.2		
2.0	12.4	42.4	43.7	−1.3	± 6	-
0.125	29.5	12.9	12.8	0.1	± 4	-
0.063	3.0	9.9	10.1	−0.2	± 2	-
	<0.063 mm	9.9	10.1	−0.2	-	±3
	<0.125 mm	12.9	12.8	0.1	-	±4
	0.063–2.0 mm	32.5	33.6	−1.1	-	±8
	≥2.0 mm	57.6	56.3	1.3	-	±8
	≥11.2 mm	0.4	0.3	0.1	-	−8 ± +5

**Table 11:** Content of voids in MAM for particular series, %.

	WC-1	WC-2	WC-3	WC-4
Content of voids in MAM, (%)	2.1	2.0	2.8	1.7

**Table 12:** Content of voids in MM and in the MM filled with binder for particular series.

	WC-1	WC-2	WC-3	WC-4
content of voids in MM (%)	15.9	15.1	15.8	15.7
content of voids in MM filled with binder, VFB (%)	86.6	86.3	82.2	89.3

**Table 13:** Value of indirect tensile strength ratio for particular series.

	WC-1	WC-2	WC-3	WC-4
ITSR (%)	97.5	95	85	112
